# Supplementary material for: Tetrodotoxins in French Bivalve Mollusks—Analytical Methodology, Environmental Dynamics and Screening of Bacterial Strain Collections
Source: Toxins (Basel). 2021 Oct 20;13(11):740. doi: 10.3390/toxins13110740 (PMC8618394; doi:10.3390/toxins13110740)
Supplement: Supplementary file 1 [file toxins-13-00740-s001.zip › toxins-1430198 Figure S1, Table S1.pdf]

Supplementary Materials: Tetrodotoxins in French Bivalve Mollusks—Analytical Methodology, Environmental Dynamics and Screening of Bacterial Strain Collections

Damien Réveillon, Véronique Savar, Estelle Schaefer, Julien Chevé, Marie-Pierre Halm-Lemeille, Dominique Hervio-Heath, Marie-Agnès Travers, Eric Abadie, Jean-Luc Rolland and Philipp Hess

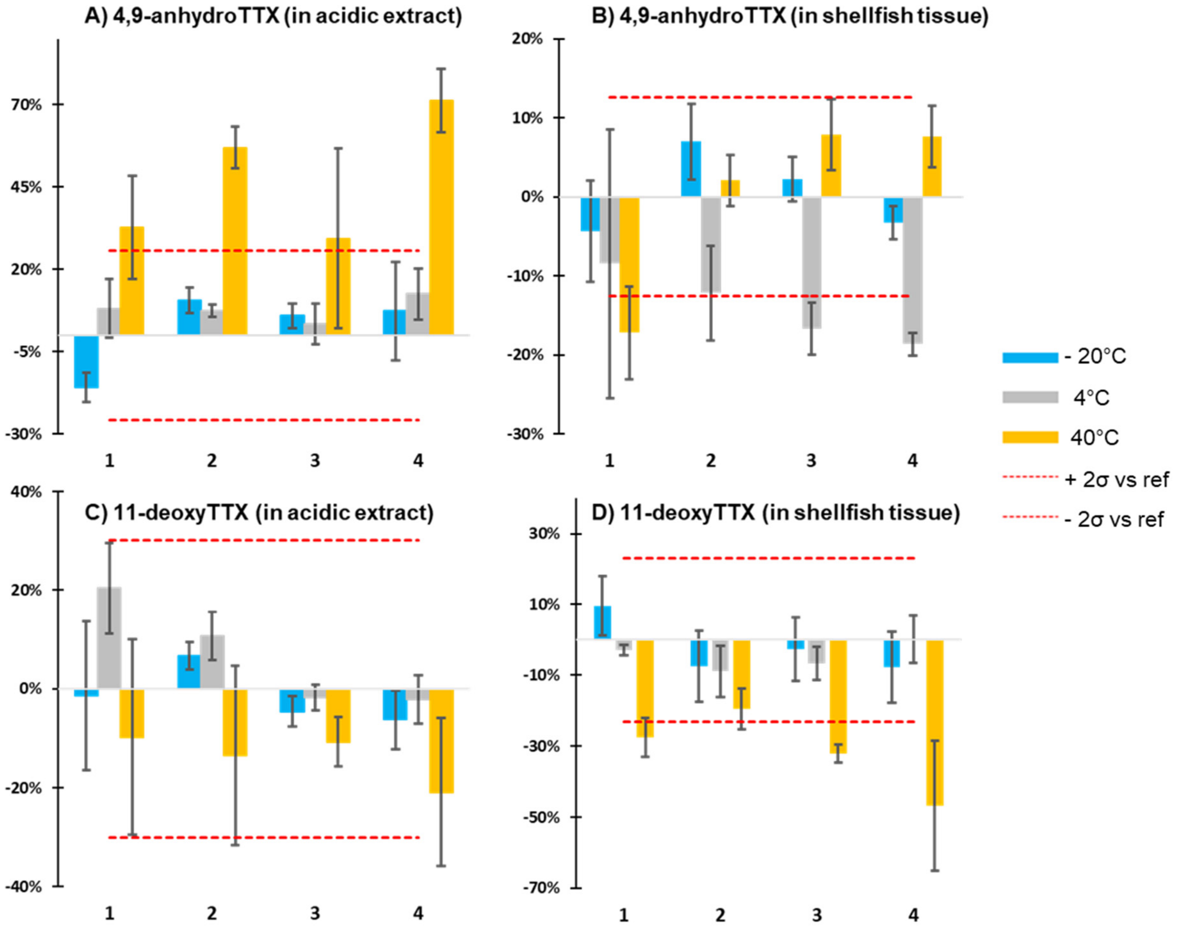

Figure S1. Four-week stability study of 4,9-anhydroTTX and 11-deoxyTTX spiked into acetic acid extract of blank oyster matrix (stored in glass vials) or blank oyster matrix at −80, −20, 4 and 40 °C. Values represent % deviations from the average of the −80 °C reference condition. (**A**) 4,9-anhydroTTX spiked into acetic acid oyster extract, (**B**) 4,9-anhydroTTX spiked into blank oyster matrix, (**C**) 11-deoxyTTX spiked into acetic acid oyster extract and (**D**) 11-deoxyTTX spiked into blank oyster matrix. Error bars represent the standard deviation (n = 3), red dotted lines delineate the confidence interval (95%, 2σ) of the −80 °C reference condition (n = 12).

Table S1. Conditions of the comparison and sensitivity (S/N) obtained using three analytical HILIC columns.

		**BEH Amide (Waters)**	**HILIC-Z (Agilent Technologies)**	**ZIC-HILIC (Merck)**
Dimensions		150 × 2.1 mm, 1.7 µm	100 × 2.1 mm, 2.7 µm	150 × 2.1 mm, 3.5 µm
Mobile phases		Turner et al, 2017 [21]	A: water + 20 mM AF + 0.1% FA<br>B: ACN + 0.1% FA	
Gradient		Turner et al, 2017 [21]	0–1 min: 70% of B<br>1–6 min: 70–50% of B<br>6–8 min: 50% of B<br>8.1 min: 70% of B<br>8.1–10 min: 70% of B	0–1 min: 60% of B<br>2–5 min: 60–50% of B<br>5–7 min: 50% of B<br>7.1 min: 60% of B<br>7.1–10 min: 60% of B
Signal-To- Noise	TTX	228	905	4209
	4-epiTTX	10	22	70
	4,9-anhydroTTX	25	103	716
	11-deoxyTTX	27	44	716
